# More about Abderhalden's Reaction

**Published:** 1914-05-30

**Authors:** 


### MORE ABOUT ABDERHALDEN'S REACTION.

On previous occasions the researches of Abderhalden, and of others who have adopted his method, have been summarised in The Hospital. The best-known application of the principle of his "reaction" is in the diagnosis of pregnancy; and enthusiastic reports have appeared from various workers as to the accuracy and delicacy of this test. Two American authors, Dr. Jellinghans and Dr. Losee, of New York, have recently published* a critical account of their experience with this reaction, which is valuable in that the limitations as well as the possibilities of the procedure are not lost sight of. They enter with great detail into all important questions of technique, and show how difficult it is to conduct the laboratory manipulations in such a way as to get consistent results. The basic principle, they say, of the test gives the impression of being very simple, but as a matter of fact the dialysation is extremely difficult. More than half the dialysers supplied by good manufacturers are found unfit for use when tested; moreover, usage spoils them, so that they must be retested every two or three weeks. Then, too, the reagents used have to be prepared with the greatest care, and there are innumerable minute errors that may spoil the result of the test.

The conclusions formulated are definite, and serve at least to show (whether others confirm them or not) that this test is only fit for highly expert bacteriologists to attempt, and not for clinicians. The authors do not deny the correctness of Abderhalden's "defensive ferment theory," but they do not admit that the dialysation method of putting it into practice is infallible. After a long experience they have succeeded in eliminating many sources of error, and their results have correspondingly improved. They now practically always get a positive result with a pregnant serum; but they still get a certain number of positives with non-pregnant sera, and this in spite of following Abderhalden's instructions implicitly. In this they are in accordance with several other American investigators; whereas in Germany there seems to be a disposition to regard the test as almost infallible. Jellinghans and Losee admit that their technique may be erroneous, but they cannot discover in what respect; inasmuch as they have several hundred cases, and have evidently taken great pains to secure the most perfect results, it seems doubtful whether their technique can really be at fault.

They lay great stress upon the unreliability of the test as a definite indication of pregnancy. Many authors, it is said, when they get a positive reaction in a case which eventually proves not to be one of pregnancy, content themselves with assuming that the placental albumin or some other substance used in the test has not been pure. Obviously, if the difficulty of getting the reagents pure is so great that the test is thereby often invalidated, it is impossible to lay any diagnostic stress upon the results of the test in any given case. In particular they ridicule the claim of another transatlantic worker that eclamptic patients give a weaker reaction than those who are healthy in this respect. They emphasise the fallacy of attempting to carry out this test by means of a ready-made pocket outfit, now being sold by some firm of American druggists. By personal trial they have convinced themselves that this outfit is useless. Finally, they enter into all the *minutiæ* of the test, which are many, and into the exact methods which they have found to answer best in its performance. In the discussion which followed the reading of Jellinghans' and Losee's paper before the New York Obstetrical Society scepticism about the test was expressed even more decisively than by those authors.

* *American Journal of Obstetrics*, April 1914, p. 593.

